# Annotations

**Published:** 1905-09-16

**Authors:** 


### ANNOTATIONS.

### Scandalous Official Apathy.

It is a weary and unprofitable task to attempt the conversion of the conscientious objector. Be a fact never so clear, he will not see it. But there is yet comfort for the citizen who places a higher value on the welfare of his fellow-men than upon his own ignorance. In the Medical Supplement to the Report of the Local Government Board for 1903–4, which has just been issued, attention is drawn to the fact that in the year with which the report deals, as in previous years, conscientious objection of parents is not the chief reason why so many children are unvaccinated. Very far from it. The conscientious objector is a creature who on the whole makes but little difference to the vaccination statistics of the country; and there are few districts where he is sufficiently numerous to be a serious impediment to the general welfare. On the whole he may be regarded as serving a very useful purpose, for his extraordinary perversity does more perhaps than anything else to draw public attention to the scandalous neglect and apathy of the responsible authorities, which is the true cause of the unvaccinated condition of the country to-day. A glance at the report referred to shows that in no single county throughout England and Wales is the law of the land strictly observed. Even as regards the Metropolis, where conscientious objection scarcely exists, in no union did the certificates of successful vaccination for 1902 attain the standard of 85 per cent. of the births, and a few unions, namely Bethnal Green, Mile End and Poplar, show less than 50 per cent. of their children to be vaccinated, although the certificates of conscientious objection do not amount to so much as one per cent. The neglect is due to official disregard of the country's laws by those whose duty it is to administer them honestly. We hope that the public will soon appreciate this oft-repeated tale, and deal with its defaulting officials according to their merits. In the meantime we are paying large annual sums in order to deal with a venomous and yet quite unnecessary epidemic. It will be a long time before England can regard small-pox with such unconcern as is possible in twice vaccinated Germany. But there is no conceivable reason why Great Britain should be quite so behindhand as it is in this matter at the present day.

### The Folly of Home Lessons.

Complaint having been made by a parent that children are sent home from all schools on Friday evening laden with work that will take the whole of Saturday, and perhaps the best part of Sunday to get through, he is told by the *Daily Telegraph*, which publishes his letter, that "it is no good complaining of hard work in a competitive world." This is cold comfort, but characteristic of the champions of the present system of elementary education. So far as the complaint has reference to schools maintained at the expense of the parents there is an easy remedy. If they are given excessive home lessons, and it is pleaded that the rules of the school must be adhered to, the children can be removed. But the parents of children who are educated in schools maintained at the expense of the public are bound to obey the provisions of the Education Act. They can only withdraw their children from school, even temporarily, on a certificate of illness from a medical man. It does not follow that the imposition of home lessons upon children of tender years should be accepted without a murmur. The Saturday holiday is an excellent institution, if the children obtain the full benefit of it, but not when it merely means a period of leisure for the teachers. The stress and strain of hard work have to be borne by the vast majority of adults; in this world the race is generally to the swift and the battle to the strong. But compulsory home lessons inflicted on boys and girls of eight or nine do not tend to equip them for the obligations of life. They are much more likely to retard their progress. They overtax the mental faculties at a time when it is particularly essential that they should not be overtaxed; they interfere with the physical development of the children, which is of vital importance; and even the most thickheaded can recognise the folly of a system which enables a boy to come out first in a competitive examination at fourteen years of age and qualifies him for a lunatic asylum at forty.

### Alcohol as a Medicine.

It is not overstating the matter to say that our grandfathers, lay and medical alike, regarded alcohol, especially in the form of spirit, as the prime resource in cases of severe illness or injury. Even to-day the majority of householders look upon the brandy-bottle as a fetish to charm away disease and death. Slowly and reluctantly, but none the less surely, this monstrous superstition is yielding in the light of modern scientific knowledge. Yesterday we were taught that shock was to be counteracted by large doses of brandy; to-day those who have studied the problem most carefully in the laboratory and by the sick-bed, and who are entitled to direct professional opinion on the matter, inform us that to administer alcohol to the individual suffering from shock is to increase the danger to his life. Thirty years ago the leaders of professional opinion in this country thought it was iniquitous to withhold alcohol from patients suffering from typhoid fever. Now, as we learn from a paper written by Dr. Dawson Burns for presentation to the International Congress against Alcoholism, which meets at Buda-Pesth this week, the London Temperance Hospital is able to show for a period of 33 years a mortality of only 14·4 per cent. in all cases of typhoid fever treated in the hospital, the mortality for the last 10 years being 12·27 per cent. The majority of these patients were not given alcohol. It will be seen that the results are not inferior to those obtained at other metropolitan institutions. For example, the mortality among cases treated in the Metropolitan Asylums Board Hospitals during the year 1904 was 14·58 per cent. We are far from being in agreement with the intemperate and wholesale condemnations of alcohol that are so constantly thrust upon us by the self-styled temperance reformer. We maintain that in moderation alcoholic drinks are pleasant and harmless. But we desire to point out that the value of alcohol and alcoholic beverages in the treatment of acute diseases is not so great as medical men have hitherto supposed.

